# Copeptin as a Marker for Severity and Prognosis of Aneurysmal Subarachnoid Hemorrhage

**DOI:** 10.1371/journal.pone.0053191

**Published:** 2013-01-11

**Authors:** Christian Fung, Gian Marco De Marchis, Mira Katan, Marleen Seiler, Marcel Arnold, Jan Gralla, Andreas Raabe, Jürgen Beck

**Affiliations:** 1 Department of Neurosurgery, Bern University Hospital, Bern, Switzerland; 2 Department of Neurology, Bern University Hospital, Bern, Switzerland; 3 Department of Neurology, College of Physicians and Surgeons, Columbia University, New York, New York, United States of America; 4 Thermo Fisher Scientific, Thermo Scientific Biomarkers, Clinical Diagnostics, Hennigsdorf, Germany; 5 Institute for Diagnostic and Interventional Neuroradiology, Bern University Hospital, Bern, Switzerland; University of Regensburg, Germany

## Abstract

**Background:**

Grading of patients with aneurysmal subarachnoid hemorrhage (aSAH) is often confounded by seizure, hydrocephalus or sedation and the prediction of prognosis remains difficult. Recently, copeptin has been identified as a serum marker for outcomes in acute ischemic stroke and intracerebral hemorrhage (ICH). We investigated whether copeptin might serve as a marker for severity and prognosis in aSAH.

**Methods:**

Eighteen consecutive patients with aSAH had plasma copeptin levels measured with a validated chemiluminescence sandwich immunoassay. The primary endpoint was the association of copeptin levels at admission with the World Federation of Neurological Surgeons (WFNS) grade score after resuscitation. Levels of copeptin were compared across clinical and radiological scores as well as between patients with ICH, intraventricular hemorrhage, hydrocephalus, vasospasm and ischemia.

**Results:**

Copeptin levels were significantly associated with the severity of aSAH measured by WFNS grade (P = 0.006), the amount of subarachnoid blood (P = 0.03) and the occurrence of ICH (P = 0.02). There was also a trend between copeptin levels and functional clinical outcome at 6-months (P = 0.054). No other clinical outcomes showed any statistically significant association.

**Conclusions:**

Copeptin may indicate clinical severity of the initial bleeding and may therefore help in guiding treatment decisions in the setting of aSAH. These initial results show that copeptin might also have prognostic value for clinical outcome in aSAH.

## Introduction

For assessment of the severity of aneurysmal subarachnoid hemorrhage (aSAH), the World Federation of Neurological Surgeons (WFNS) grade is the current gold standard [1]. The condition of patients with aSAH may however be confounded by seizure, hydrocephalus or sedation [2]. Treatment decisions such as occlusion of an aneurysm are based on clinical grading [1]. Prediction of outcome remains difficult and complicates decision-making for active treatment [3]. Copeptin has evolved as a serum marker for the severity of disease and outcome of patients in spontaneous intracerebral hemorrhage (ICH), ischemic stroke and sepsis [4], [5], [6]. It is released in an equimolar ratio with vasopressin from a common precursor protein, pre-provasopressin [7]. Vasopressin is a hypothalamic hormone that is stimulated by different stressors. Vasopressin potentiates the action of corticotrophin releasing hormone and leads downstream to a release of adrenocorticotropic hormone (ACTH) and production of cortisol [8]. Copeptin is a 39 amino-acid glycopeptide and is equivalent to the C-terminal part of pre-provasopressin [7]. Because copeptin is stable for days, it can be easily measured in blood samples [7]. Copeptin therefore is a surrogate marker for vasopressin release and might act as a marker for stress response [7]. The aim of this prospective study was to elucidate whether copeptin could be used as a marker for severity and prognosis of aSAH.

## Materials and Methods

Patients with aSAH admitted to our neurosurgical unit from 21 December 2010 through 21 March 2011 were prospectively included. The study was approved by the Bern Canton Ethics Committee (Bern, Switzerland) and written informed consent was obtained from all patients (Clinical Trial Registration Information NCT00878813).

We collected demographic, clinical, radiological and outcome data for all patients. Venous blood was collected in ethylenediaminetetraacetic acid (EDTA) tubes on admission and a sample was analyzed for copeptin.

Samples were placed on ice, centrifuged at 3000 g, and plasma aliquoted and frozen at −70°C. We analyzed copeptin levels using a validated commercial chemiluminescence sandwich immunoassay (Thermo Scientific B.R.A.H.M.S LIA CT-proAVP, B.R.A.H.M.S GmbH, Henningsdorf, Germany) [5].

For grading, the best Glasgow Coma Scale (GCS) after resuscitation in unsedated patients was used [1], [9]. Imaging at admission was used to determine Fisher grade, volume of ICH and hydrocephalus [10], [11]. Patients were divided into 2 groups: those with diffuse subarachnoid blood (Fisher grade 2 and 4), and patients with thick subarachnoid blood clot (Fisher grade 3). Admission CT angiography or digital subtraction angiography was used for aneurysm detection. Aneurysm location was divided into those originating from the anterior and posterior circulation [12]. Cerebral vasospasm and ischemic lesions were defined using the CONSCIOUS criteria [9]. Ischemic lesions were stratified into small (<10 mL), medium (10–100 mL) and large (>100 mL). Clinical outcome was evaluated at the 6-month follow-up visit and categorized according to the patient's modified Rankin scale (mRS) score: a good outcome was defined as mRS 0–3 and a poor outcome as mRS 4–6.

The primary endpoint was the association of admission copeptin levels with WFNS grade. Fisher grade, ICH, intraventricular hemorrhage (IVH), hydrocephalus, vasospasm, ischemia and mortality were defined as secondary endpoints.

Comparisons of copeptin levels with clinical scales and endpoints were calculated using an unpaired Mann-Whitney-*U* test (StataCorp, College Station, TX, USA). Two-sided P-values<0.05 were considered significant.

## Results

**Figure 1:**
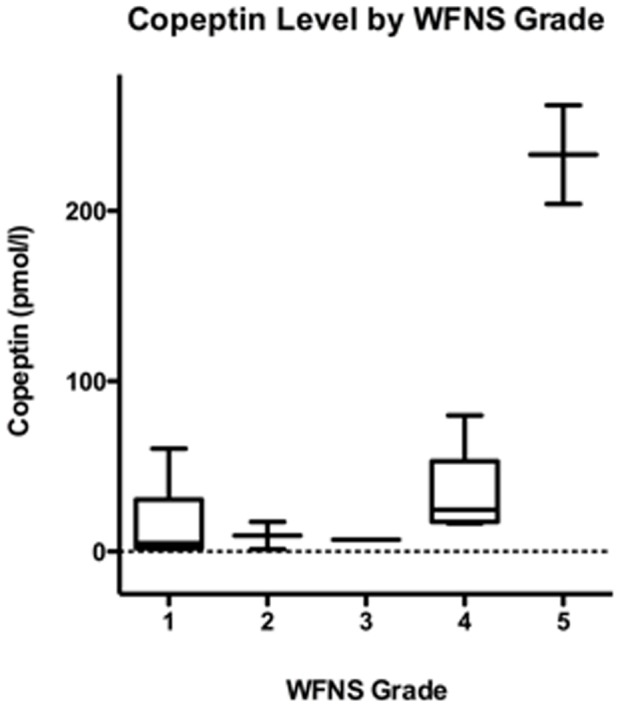
Copeptin Level by WFNS Grade. The figure represents a boxplot of copeptin values per WFNS grade. Displayed are lowest, highest and median copeptin values (pmol/L), with upper (75%) and lower (25%) quartiles.

Our study included a total of 18 patients (12 women and 6 men), and the median age was 57 years (interquartile range (IQR) 48–67 years). Copeptin values and distributions are shown in Table 1 and Fig. 1. The overall median copeptin level at admission was 17.0 pmol/L (IQR 3.3–38.4). The median copeptin level was 6.8 pmol/L for patients WFNS 1 (n = 8), 2.8 pmol/L for WFNS 2 (n = 2), 7.1 pmol/L for WFNS 3 (n = 1), 17.4 pmol/L for WFNS 4 (n =  5) and 79.9 pmol/L for WFNS 5 (n = 2). Median intraparenchymal hematoma volume was 27.5 mL. Three patients developed ischemic lesions, all of which were assessed as small (<10 mL).

**Table 1:** Copeptin levels and characteristics of study patients.

	n	Median copeptin level (IQR) (pmol/L)	p-value
**WFNS grade** a			
Good-grade	11	6.8 (6.6–17.4)	
Poor-grade	7	26.3 (18.3–204)	**P = 0.006**
**Fisher grade**			
Diffuse blood (Fisher grade 2 and 4)	3	1.6 (1.5–3.3)	
Thick clot (Fisher grade 3)	15	18.3 (6.8–60.4)	**P = 0.027**
**ICH**			
Yes	6	52.5 (17.4–204)	
No	12	6.7 (2.2–22.3)	**P = 0.02**
**IVH**			
Yes	11	17.4 (6.6–38.4)	
No	7	7.1 (1.6–79.9)	P = 0.89
**Hydrocephalus**			
Yes	12	17.9 (5–32.4)	
No	6	6.7 (3.3–60.4)	P = 0.682
**Vasospasm**			
Yes	5	26.3 (24.5–60.4)	
No	13	7.1 (2.8–18.3)	P = 0.15
**Ischemia**			
Yes	3	24.5 (1.3–60.4)	
No	15	16.5 (3.3–38.4)	P = 1.0
**Sex**			
Male	6	21.4 (12.7–94.3)	
Female	12	7 (2.9–51.9)	P = 0.5
**Aneurysm location**			
Anterior circulation	11	17.4 (6.8–60.4)	
Posterior circulation	4	2.2 (1.4–29.5)	P = 0.38
**Outcome** b			
Good	13	6.8 (2.8–24.5)	
Poor	5	26.3 (17.4–204)	P = 0.054
**Mortality**			
Survivors	14	7.0 (2.8–38.4)	
Deceased	4	21.9 (17–115.2)	P = 0.277

IQR: interquartile range; WFNS: World Federation of Neurological Surgeons; ICH: intracerebral hemorrhage; IVH: intraventricular hemorrhage.

a WFNS grade: Good-grade WFNS 1–3; Poor-grade: WFNS 4–5.

b modified Rankin Scale (mRS) score 0–3: Good outcome; mRS 4–6: Poor outcome.

Higher copeptin levels were significantly associated with poor-grade (WFNS 4–5) aSAH, Fisher grade 3 and ICH (Table 1). 15 out of 18 patients had a proven aneurysm. Among the remaining 3 patients, one had a perimesencephal SAH, one died before an aneurysm could be detected, and in one patient – although having a SAH Fisher grade 3 – the aneurysm could not be proven. 11 aneurysms were located in the anterior and 4 in the posterior circulation. Aneurysm location was not significantly associated with copeptin levels (P = 0.38; Table 1). In addition, sex was not significantly associated with copeptin levels; the 6 male patients had a median copeptin level of 21.4 pmol/L compared to 7 pmol/L for the 12 female patients (Table 1). Copeptin levels in patients with a good outcome were lower than those with a poor outcome (P = 0.054, Table 1). Patients that died after aSAH had median copeptin levels three times higher than aSAH survivors at 6-months (P = 0.277).

## Discussion

The main finding of the current study is that copeptin reflects the severity of aSAH, as assessed by the WFNS score after resuscitation and similarly to the grading of the severity of ischemic stroke [4]. The median copeptin level of our study cohort was comparable to reported median levels in cohorts with hemorrhagic stroke (16.3 pmol/L) and ischemic stroke (11.6 pmol/L) [4], [6]. Median copeptin levels in healthy individuals have been reported, and range from 3.7 to 4.2 pmol/L [4], [7], [13]. In the current study, the presence of ICH in patients with aSAH was statistically significantly associated with higher levels of copeptin. Zweifel et al. showed that higher levels of copeptin in patients with ICH were associated with increased hematoma volume and poorer clinical outcome [6]. We have now demonstrated a similar relationship in aSAH patients; increased copeptin is significantly associated with the amount of subarachnoid blood. Due to the fact that the majority of poor grade SAH patients also display an ICH, the data must be interpreted with caution. Whether high levels of copeptin can be attributed solely to the severity of the SAH as rated by the WFNS grade or also to the occurrence of ICH cannot be answered. Yet in general, the occurrence of ICH has an impact on the severity of the clinical picture and outcome of SAH patients. We found no statistically significant association of copeptin with IVH, hydrocephalus, vasospasm, aneurysm location, sex, delayed ischemia, or mortality within 6 months, although there was a trend towards an association with functional outcome.

Delayed cerebral vasospasm is a well-recognized contributor to poor outcome [2], [9]. The cascade of neurovascular events encompassing changes early after the ictus as well as delayed cerebral ischemia due to vasospasm determines final outcome. So far, biomarkers on admission fail to reliably predict delayed cerebrovascular events [14]. In our opinion admission copeptin levels might not predict later cerebrovascular events, yet serial testing throughout the course of the disease might show an association. Still, in a recent retrospective analysis, Zhu et al. found a significant association between copeptin and vasospasm [15]. However, in line with our current results, they concluded that admission copeptin levels should not be used as a predictor for vasospasm because clinical grades had a higher accuracy [15].

Whether copeptin release is SAH specific or reflects a general stress response remains unclear. Copeptin is released from the hypothalamus in an equimolar ratio with vasopressin. The increase of copeptin in SAH patients might therefore rather result from activation of the hypothalamo-pituitary-adrenal axis as a response to stress [4]. This is very likely since, besides ischemic and hemorrhagic stroke, this has been shown for various diseases like respiratory tract infection, heart failure and shock [5], [16], [17]. Still, this does not exclude the role of copeptin as a potential biomarker for severity of SAH.

Despite the strength of the prospective protocol and the detailed patient characterization, our pilot study was too small to draw definitive conclusions. However the results of this study, as well as the similar findings by Zhu et al. [15], should encourage further investigation on copeptin in patients with aSAH.

### Conclusions

Copeptin levels on admission were statistically significantly associated with severity of aSAH according to the WFNS grade, the amount of subarachnoid blood and ICH. Copeptin levels tended to be higher in patients with unfavorable clinical outcome. Copeptin might therefore evolve as a useful marker for prognosis in subarachnoid hemorrhage.
